# Measuring *in vivo* responses to endogenous and exogenous oxidative stress using a novel haem oxygenase 1 reporter mouse

**DOI:** 10.1113/JP274915

**Published:** 2017-11-23

**Authors:** Michael McMahon, Shaohong Ding, Lourdes P. Acosta‐Jimenez, Tania G. Frangova, Colin J. Henderson, C. Roland Wolf

**Affiliations:** ^1^ School of Medicine, Ninewells Hospital and Medical School University of Dundee Dundee DD1 9SY UK

**Keywords:** oxidative stress, gene expression, heme oxygenase 1

## Abstract

**Key points:**

- Haem oxygenase 1 (Hmox1) is a cytoprotective enzyme with anti‐inflammatory and anti‐oxidant properties that is induced in response to multiple noxious environmental stimuli and disease states.
- Tools to enable its expression to be monitored *in vivo* have been unavailable until now.
- In a new Hmox1 reporter model we provide high‐fidelity, single‐cell resolution blueprints for Hmox1 expression throughout the body of mice.
- We show for the first time that Hmox1 is constitutively expressed at barrier tissues at the interface between the internal and external environments, and that it is highly induced in muscle cells during systemic inflammation.
- These data suggest novel biological insights into the role of Hmox1 and pave the way for the use of the model to study the role of environmental stress in disease pathology.

**Abstract:**

Hmox1 protein holds great promise as a biomarker of *in vivo* stress responses as it is highly induced in stressed or damaged cells. However, Hmox1 expression patterns have thus far only been available in simple model organisms with limited relevance to humans. We now report a new Hmox1 reporter line that makes it possible to obtain this information in mice, a premiere model system for studying human disease and toxicology. Using a state‐of‐the‐art strategy, we expressed multiple complementary reporter molecules from the murine *Hmox1* locus, including firefly luciferase, to allow long‐term, non‐invasive imaging of Hmox1 expression, and β‐galactosidase for high‐resolution mapping of expression patterns post‐mortem. We validated the model by confirming the fidelity of reporter expression, and its responsiveness to oxidative and inflammatory stimuli. In addition to providing blueprints for Hmox1 expression in mice that provide novel biological insights, this work paves the way for the broad application of this model to establish cellular stresses induced by endogenous processes and those resulting from exposure to drugs and environmental agents. It will also enable studies on the role of oxidative stress in the pathogenesis of disease and its prevention.

## Key points

- Haem oxygenase 1 (Hmox1) is a cytoprotective enzyme with anti‐inflammatory and anti‐oxidant properties that is induced in response to multiple noxious environmental stimuli and disease states.
- Tools to enable its expression to be monitored *in vivo* have been unavailable until now.
- In a new Hmox1 reporter model we provide high‐fidelity, single‐cell resolution blueprints for Hmox1 expression throughout the body of mice.
- We show for the first time that Hmox1 is constitutively expressed at barrier tissues at the interface between the internal and external environments, and that it is highly induced in muscle cells during systemic inflammation.
- These data suggest novel biological insights into the role of Hmox1 and pave the way for the use of the model to study the role of environmental stress in disease pathology.

## Introduction

Adaption to environmental stress leading to cell survival represents a fundamental property of all living organisms (Kultz, 2005; Gasch, 2007). A wide range of stress‐inducible response pathways have evolved including those activated by heat‐shock, DNA damage, chemical toxicity and oxidative stress (Kensler *et al*. 2007). Typically, these stress responses are transiently induced in organisms exposed to toxic substances and often result in the stalling of normal metabolic processes, such as the cell cycle, to allow the repair of damage to occur. These pathways are activated by exposure to environmental agents and by endogenous metabolic pathways such as mitochondrial respiration (Schulz *et al*. 2007). In addition there is strong evidence that these pathways are chronically activated as part of the aetiology of degenerative disease (Ames *et al*. 1993). This has been formally confirmed for progeroid syndromes (Carrero *et al*. 2016). These adaptive response genes provide biomarkers of organismal homeostasis and health (Rand, 2010).

Significant insights into the armamentarium of stress response proteins and how they are regulated have been made in studies in simple organisms such as *Escherichia coli*, *Saccharomyces cerevisiae*, *Drosophila melanogaster* and *Caenorhabditis elegans* (Morin *et al*. 2001; Newman *et al*. 2006; Zaslaver *et al*. 2006; Hunt‐Newbury *et al*. 2007). However, our understanding of these pathways in mammals *in vivo* is much more limited. This is because of the complexities of *in vivo* experiments in mice and rats and also the lack of experimental tools to measure what are often transient responses *in vivo*.

One approach to solve this conundrum has been the development of transgenic reporter mice. In this approach, a reporter gene driven by a stress‐inducible promoter is inserted into the mouse genome. Originally, reporters driven off variable lengths of a gene promoter were randomly integrated into the genome. This approach had a number of limitations particularly because, firstly, the length of the gene promoter required to get *bone fide* recapitulation of the endogenous gene expression pattern is unknown, and secondly, the site of integration of the reporter construct can also result in aberrant patterns of transgene expression (Gong *et al*. 2003). This problem was to a degree addressed by inserting the reporter element into the endogenous gene locus. This strategy has been used generically to create large collections of mouse reporter strains as part of the International Mouse Phenotyping Consortium (Skarnes *et al*. 2011). Yet this strategy still has some substantial drawbacks caused by the loss of some or all of the target gene sequence during insertion of the reporter cassette. First, the resulting loss of one allele raises concerns regarding haplo‐insufficiency effects. Second, the lost sequences typically contribute to target protein expression by governing transcript stability and translatability (Dreyfuss *et al*. 2002; Matsui *et al*. 2007; Nilsen, 2007). This layer of gene control is therefore lost at the reporter allele, which is unlikely as a consequence to accurately recapitulate target protein expression patterns. Finally, this strategy had the limitation that the use of multiple reporters was not supported, restricting the versatility of the resulting models (Martin *et al*. 2009). To circumvent these issues, we have exploited the use of viral 2A technology (de Felipe *et al*. 2006) which allows the expression of multiple proteins from a single gene locus while retaining all target gene sequences. We have used this technology to generate mouse reporter lines that allow the *in vivo* measurement of cellular responses to stress both in real time and also at single cell resolution (McMahon *et al*. 2016).

Haem oxygenase 1 (Hmox1) is a centrally important cytoprotective enzyme found in most species. It catalyses the rate‐limiting step in the metabolic degradation of free haem to bile pigments (biliverdin and bilirubin), carbon monoxide and iron (Maines, 1997). In healthy animals, its expression is restricted to macrophages of the spleen and other tissues that degrade senescent red blood cells, such as the liver and bone marrow (Abraham & Kappas, 2008). However, it is induced in almost all cell types *in vitro* and *in vivo* in response not only to its natural substrate haem but to a wide range of environmental stressors and disease states (Keyse & Tyrrell, 1989; Poss & Tonegawa, 1997a; Soares & Bach, 2009). Induction of Hmox1, almost without exception, is considered to be cyto‐protective, which is generally ascribed to its ability to convert haem to carbon monoxide and bilirubin, the first product being anti‐inflammatory, and the latter anti‐oxidant (Gozzelino *et al*. 2010).

The almost ubiquitous induction of *Hmox*1 in response to multiple forms of stress is a consequence of proximal promoter and distal enhancers in the gene for distinct classes of stress‐activated transcription factors. The most important of these transcription factors are NFE2‐related factor‐2 (Nrf2) and its paralogues, and members of the heat shock factor (Hsf), nuclear factor kappa‐light‐chain‐enhancer of activated B cells (NF‐κB), and metal regulatory transcription factor 1 (Mtf1) families of proteins (Ryter *et al*. 2006). Another critical transcription factor, BTB and CNC homology 1 protein (Bach1), represses *Hmox1* expression by competing with Nrf2 for binding to the *Hmox1* distal enhancer (Sun *et al*. 2004). As a consequence of input from all these transcription factors, *Hmox1* gene expression provides a highly sensitive sensor for a range of cellular stress responses and an attractive gene for the development of an *in vivo* reporter system to assess the role of oxidative stress and other stresses in toxicology and in the pathogenesis of diseases where such stresses have been implicated (Ryter *et al*. 2006).

The only *Hmox1* reporter mice described to date, a line developed by us, were created by random integration of either a luciferase (Malstrom *et al*. 2004) or β‐galactosidase (Young *et al*. 2010) under the control of approximately 15 kb of *Hmox1* promoter. In this paper, we have used viral 2A technology to develop a new Hmox1 reporter mouse where multiple reporters are expressed off the endogenous *Hmox1* gene promoter. Using this model we have mapped at high‐resolution the expression of Hmox1 in healthy mice revealing novel biological insights into the functions of this protein. In addition to studying the regulation of Hmox1, we also demonstrate how this model can be used to study toxicological processes. Future work will demonstrate how the model can be used to study and find new treatments for degenerative disease.

## Methods

### Ethical approval

All animal work described in this study was approved by the Welfare and Ethical treatment of Animals Committee of the University of Dundee. Those carrying out animal work in this study did so with Personal and Project Licences granted by the UK Home Office under the Animals (Scientific Procedures) act (1986), as amended by EU Directive 2010/63/EU. Animals were inspected regularly by staff trained and experienced in small animal husbandry, with 24 h access to veterinary advice. Investigators were familiar, and all experiments complied with, the ethical principles of *The Journal* as outlined by Grundy (2015).

### Animals

All animals were supplied from the Medical School Resource Unit, University of Dundee, on a C57BL/6NTac background, and genetically manipulated to insert reporter genes into either the p21 locus (McMahon *et al*. 2016) or the *Hmox1* locus (this study, see below for further details). Mice were maintained in Tecniplast Sealsave micro‐isolator cages containing Eco‐Pure chip7D (Datesand Group, Manchester, UK) for bedding with *ad libitum* access to food (RM1, Special Diet Services, Stepfield, Witham, Essex, UK) and water, and a 12 h light/dark environment. Temperature and relative humidity were maintained between 20°C and 24°C, and 45% and 65%, respectively.

Anaesthesia was induced in an anaesthetic rig by gaseous isoflurane – 2 l min^−1^ oxygen and 3.5–4.0% isofluorane for induction. Mice were transferred to the Caliper IVIS Lumina II imager (PerkinElmer, Llantrisant, UK) and anaesthesia maintained (1.5 l min^−1^ oxygen, 1.5–2.5% isofluorane) for the duration of *in vivo* imaging by use of gas anaesthesia ports and a five‐position manifold within the instrument. After imaging, animals were removed from the Lumina II instrument, and allowed to recover in an open‐top cage, with constant monitoring.

At the end of studies, all animals were killed according to Schedule 1 of the Animals (Scientific Procedures) Act (1986) by exposure to a rising concentration of CO_2_ and death confirmed by exsanguination.

### Study design

Data in this paper were obtained using mice of both sexes and between 14 and 41 weeks of age, as specified in individual figure legends. In any given experiment, mice were age‐matched to within 1 month of each other, unless otherwise stated. Animals were randomly assigned to control or treatment groups using the Mersenne Twister random number generator in the R programming environment. Analysts were not blinded to the identity of biological samples.

### Hmox1 reporter mice

#### Construction

For the generation of Hmox1 reporter mice, a DNA cassette encoding an open reading frame for T2A‐LacZ‐T2A‐hCG‐T2A‐Fluc (T2A, 2A peptide sequence from *Thoseaasigna* virus; LacZ, β‐galactosidase; hCG, human chorionic gonadotropin; FLuc, firefly luciferase, codon‐optimized for expression in mammalian cells) was inserted between the penultimate and STOP codons in exon 5 of *Hmox1*. The positive selection marker (puromycin resistance (*PuroR*)) was flanked by *FRT* sites to allow for removal after the successful generation of transgenic mice. The targeting vector was generated using bacterial artificial chromosome (BAC) clones from the C57BL/6J RPCIB‐731 BAC library and electroporated into TaconicArtemis C57BL/6NTac ES cell line Art B6/3.6. Positive clones were verified by PCR and Southern blot before being injected into blastocysts from super‐ovulated BALB/c mice. Blastocysts were injected into pseudo‐pregnant NMRI females, and the chimerism of offspring was evaluated by coat colour. Highly chimeric mice were bred with C57BL/6 females mutant for the gene encoding Flp recombinase (C57BL/6‐Tg(CAG‐Flpe)2 Arte). Germline transmission was identified by the presence of black C57BL/6 offspring (G1). The gene encoding Flp was removed by further breeding to Flp partners after successful verification of *PuroR* removal.

#### Genotyping

Ear biopsies of mice 4–8 weeks old were incubated at 50°C for 4–5 h in lysis buffer containing 75 mm NaCl, 25 mm EDTA, 1% (w/v) SDS and 100 μg ml^−1^ (39 U mg^−1^) proteinase K (Sigma). The concentration of NaCl in the reaction was raised to 0.6 m and a chloroform extraction was performed. Two volumes of isopropyl alcohol were added to the extracted supernatant to precipitate genomic DNA (gDNA). A 40 μl volume of TE buffer (10 mm Tris, 1 mm EDTA, pH 8.0) was added to the pellet, and subsequently gDNA was dissolved overnight at 37°C. The typical PCR sample consisted of a 25 μl volume containing 10 pmol of the primers (6395_29 5′‐GCTGTATTACCTTTGGAGCAGG‐3′; 6395_30 5′‐CCAAAGAGGTAGCTAATTCTATCAGG‐3′). Each reaction also contained 1.25 U *Taq* DNA polymerase (Thermo‐Scientific) with 10 mm dNTPs, buffer and 25 mm MgCl_2_. The following PCR conditions were applied: 5 min, 95°C initial denaturation; 30 s, 95°C cyclic denaturation; 30 s, 60°C cyclic annealing; 1 min, 72°C cyclic elongation for a total of 35 cycles, followed by a 10 min 72°C elongation step. All PCR protocols were developed by TaconicArtemis. PCR amplification products were analysed by agarose gel electrophoresis.

### p21 reporter mice

These mice and accompanying genotyping protocols have previously been reported (McMahon *et al*. 2016). Briefly, these mice contain a DNA cassette encoding an open reading frame for T2A‐LacZ‐T2A‐Fluc inserted between the penultimate and STOP codons of the *CDKN1A* (*p21*) gene. The mouse expresses both β‐galactosidase and firefly luciferase (codon‐optimized for mammalian cells) in amounts that parallel that of endogenous p21.

### Chemicals and γ‐irradiation

All chemicals were from Sigma. Haemin was dissolved in 0.1 m NaOH prior to adjusting pH to 7.4 with 1 m HCl. Cadmium chloride and lipopolysaccharide (LPS) from *Escherichia coli* strain O55:B5 were both prepared in 0.9% (w/v) NaCl. Solvent was used as vehicle control in all mouse experiments. Butylated hydroxyanisole (BHA) powder was administered in the foodstuff. Mice were γ‐irradiated in an Oris IBL 637 caesium‐137 irradiator (3 min Gy^−1^).

### *In vivo* luciferase imaging

Imaging was performed at baseline and after chemical dosing and/or irradiation. Reporter mice were injected i.p. with 5 μl g^−1^ body weight RediJect d‐Luciferin (30 mg ml^−1^, Caliper) and anaesthetized by isofluorane before being transferred into the IVIS Lumina II imaging chamber (Caliper) for bioluminescence imaging. Luminescent images (5 s, f/stop 1.2, binning 4) and greyscale images (2 s, f/stop 16, binning 2) were acquired. Photon fluxes in regions of interest (ROIs) were quantified using the LivingImage (R) Software, version 4.3.1 (Caliper). ROIs were defined as an oval from just below the forepaws to just above the genitals of each mouse. Photon fluxes are expressed as photons s^−1^ cm^−2^ sr^−1^. Luminescent images were rendered using the fire look‐up table in ImageJ, and superimposed on greyscale photographs.

### *In vitro* luciferase activity assay

Luciferase activity was measured using the Luciferase Assay System (Promega), according to the manufacturer's instructions. Briefly, whole‐cell lysates were prepared from flash‐frozen organs as follows. The organ was pulverized under liquid nitrogen with a mortar and pestle. The powder was added to radioimmunoprecipitation assay (RIPA) buffer (25 mm Tris (pH 7.4), 150 mm NaCl, 0.1% (w/v) SDS, 0.5% (w/v) deoxycholic acid, 1% (v/v) Igepal‐600) supplemented with Roche cOmplete EDTA‐free tablets (Sigma‐Aldrich, Dorset, UK) and vortexed vigorously. After 30 min on ice, the resulting lysate was sonicated to reduce viscosity, clarified by centrifugation (16,000 *g*, 10 min, 4°C) and protein concentrations in the resulting supernatant were determined using the ‘Microplate BCA Protein Assay Kit – Reducing Agent Compatible’ from Thermo Scientific, according to the manufacturer's instructions. Protein concentrations in the supernatant were equalized to 30 mg ml^−1^ by addition of RIPA buffer. A 5 μl portion of this supernatant was mixed with 25 μl of Luciferase Assay Reagent and the luminescence was quantified using the Orion II Microplate Luminometer (Berthold Detection Systems).

### Tissue harvesting and processing for cryo‐sectioning

Mice were killed by exposure to rising concentrations of CO_2_. The median lobe of the liver was fixed in 10% neutral buffered formalin. The proximal 2–4 cm of duodena were fixed in 4% (w/v) paraformaldehyde. All other organs, including sagittally cut kidney, the stomach, the proximal 2 cm of the large intestine, the thymus, the spleen, the left lung lobe, the heart and the brain were fixed in Mirsky's fixative (National Diagnostics). Tissues were stored at 4°C, formalin‐fixed tissues for 4 h, Mirsky‐fixed tissues overnight, before being transferred into 30% (w/v) sucrose for 24 h. Embedding was carried out in Shandon M‐1 Embedding Matrix (Thermo Scientific) in a dry ice–isopentane bath. Sectioning was performed on an OFT5000 cryostat (Bright Instrument Co.). With the exception of lung and brain sections, all sections were cut at 10 μm thickness with a chamber temperature of −20°C. Lung sections were cut at 12 μm thickness with a chamber temperature of −23°C. Brain sections were cut at 20 μm thickness with a chamber temperature of −23°C.

### *In situ* β‐galactosidase (β‐gal) staining

Sections were thawed at room temperature and rehydrated in PBS supplemented with 2 mm MgCl_2_ for 5 min before being incubated overnight at 37°C in X‐gal staining solution (PBS (pH 7.4) containing 2 mm MgCl_2_, 0.01% (w/v) sodium deoxycholate, 0.02% (v/v) Igepal CA630, 5 mm potassium ferricyanide, 5 mm potassium ferrocyanide and 1 mg ml^−1^ 5‐bromo‐4‐chloro‐3‐indolyl β‐d‐galactopyranoside). On the following day, slides were washed in PBS, counterstained in Nuclear Fast Red (Vector Laboratories) for 5 min, washed twice in distilled water and dehydrated through 70% and 95% ethanol before being incubated in Histoclear (VWR) for 3 min, air‐dried and mounted in DPX mountant (Sigma). Slides were digitized at 40× magnification using an Aperio digital scanner (Leica) and TIF images were extracted using ImageScope software (Leica). The resulting images were rendered in ImageJ.

### Tissue harvesting and processing for Perls Prussian Blue staining

Mice were killed by exposure to rising concentrations of CO_2_. Organs were fixed in Gurr buffer overnight before transfer into 70% (v/v) ethanol. The following day, organs were dehydrated and embedded in paraffin and subsequently sectioned at 5 μm thickness using a Shandon Finesse 325 microtome.

### Perls' Prussian Blue staining

Sections were deparaffinized in xylene and rehydrated through a graded series of 100–50% (v/v) ethanol solutions, followed by immersion in distilled water. Sections were incubated for 20 min at room temperature in a freshly prepared aqueous solution containing 10% (v/v) HCl and 5% (w/v) potassium ferrocyanide. Sections were washed in distilled water, counterstained in Nuclear Fast Red for 5 min, washed in distilled water, dehydrated through 95% (v/v) ethanol and two changes of 100% (v/v) ethanol, cleared with two changes of xylene, air‐dried, and mounted in DPX medium (Sigma).

### Immunoblots

Whole‐cell lysates were prepared from flash‐frozen organs as follows. Briefly, the organ was pulverized under liquid nitrogen with a mortar and pestle. The powder was added to Laemmli sample buffer (13 mm Tris (pH 6.8), 11% (v/v) glycerol, 0.44% (w/v) SDS, 0.02% (w/v) bromophenol blue, 1.1% (v/v) 2‐mercaptoethanol) supplemented with Complete EDTA‐free protease inhibitors (Millipore) and Halt phosphatase inhibitors (Thermo Scientific) and vortexed vigorously. After 30 min on ice, the resulting lysate was sonicated to reduce viscosity and protein concentrations were determined using the ‘Microplate BCA Protein Assay Kit – Reducing Agent Compatible’ from Thermo Scientific, according to the manufacturer's instructions. SDS/polyacrylamide gel electrophoresis and immunoblotting were carried out as previously described (McMahon *et al*. 2003). Antibodies used included mouse monoclonal antibodies raised against rabbit glyceraldehyde‐3‐phosphate dehydrogenase (clone GAPDH_71.1 (Sigma catalogue number G8795)), a synthetic peptide sequence (SGPSIVHRKCF) corresponding to the C‐terminus of β‐actin (clone AC‐40 (Sigma catalogue number A4700)) and *E. coli* β‐galactosidase (clone not specified (Promega catalogue number Z3781)), rabbit polyclonal antibodies raised against rat Hmox1 (Abcam catalogue number ab13243)), goat polyclonal antibodies against firefly luciferase (Promega catalogue number G7451). Rabbit polyclonal antibodies raised in‐house against mouse Gsta1 and rat Gstm1 have been previously described (McMahon *et al*. 2001).

### Relative quantification of mRNA species

This was carried out by TaqMan chemistry. Total RNA was isolated from cells using the RNeasy Kit (Qiagen), according to the manufacturer's instructions. Approximately 1.0 μg of total RNA was reverse‐transcribed to cDNA using the QuantiTect Kit (Qiagen), according to the manufacturer's instructions. The PCR mixes were prepared by mixing 1.5 μl of cDNA with 1 μl of TaqMan probe set, 10 μl of Universal PCR Master Mix (PerkinElmer Applied Biosystems) and 7.5 μl of Milli‐Q grade water. For the real‐time PCR analysis, the following pre‐designed TaqMan probe sets in solution were used: Mn00516006_m1 (Hmox1); Hs03003631 (18s ribosomal RNA) (all from PerkinElmer Applied Biosystems). A custom TaqMan probe set designed against the β‐galactosidase sequence used in the reporter cassette was used to measure transcript produced specifically from the reporter allele (proprietary probe and primer sequences, PerkinElmer Applied Biosystems). Data acquisition and analysis utilized the ABI PRISM 7700 sequence detection system (PerkinElmer Applied Biosystems). The relative gene expression levels in different samples were calculated using the Comparative C_T_ Method as outlined in the ABI PRISM 7700 Sequence Detection System User Bulletin no. 2. The expression of 18s rRNA was used as the internal control.

### Clinical chemistry

Terminal bleeds were collected in heparinized blood collection tubes (Sarstedt). Clinical chemistry assays were performed blind at the clinical pathology laboratory, MRC Harwell (http://www.har.mrc.ac.uk/services/pathology/clinical-chemistry).

### Statistics

To test the hypothesis that a given test substance increases bioluminescence above that observed in vehicle‐treated animals we used two‐way ANOVA with repeated measures, under the assumption of normality. To test the hypothesis that *N*‐acetyl cysteine (NAC) reduces the toxicity of acetaminophen (APAP), using alanine aminotransferase (ALT) and aspartate aminotransferase (AST) blood chemistry results as endpoints, we used a 2 × 2 factorial experimental design and analysed the data by two‐way ANOVA, under the assumption of normality. The statistical tests were run under the R programming environment. The experimental unit was considered the individual animal and in general three animals were used for both control and treatment groups.

## Results

### A new model for monitoring Hmox1 expression at single‐cell resolution

**Figure 1:**
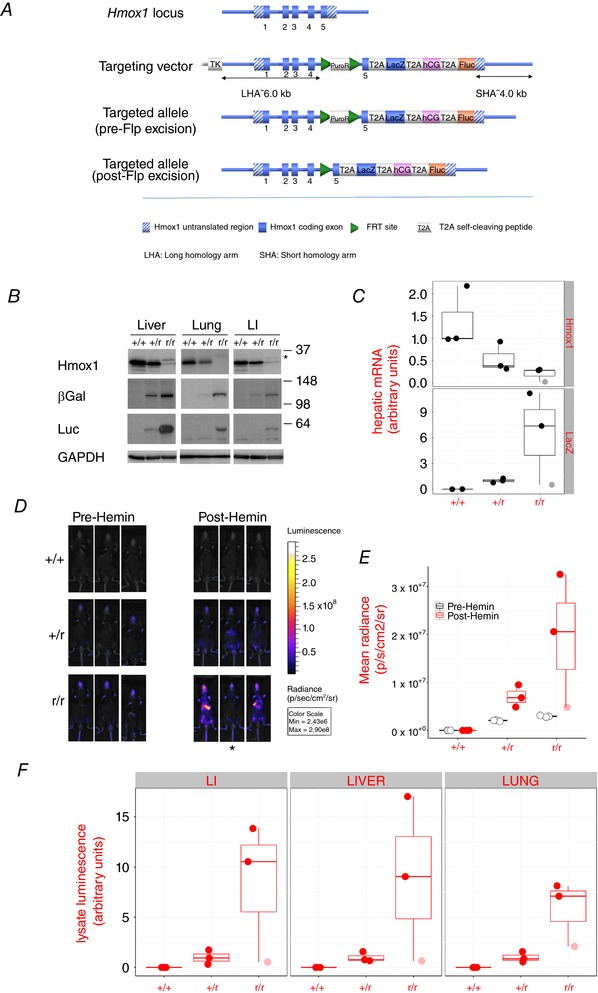
Use of T2A sequences to express β‐galactosidase, luciferase and human chorionic gonadotropin (hCG) from the endogenous *Hmox1* promoter *A*, schematic diagram of the engineering strategy used to create the *Hmox1* multireporter allele. See text for further details. *B* **–** *F*, triplicate WT (+/+), heterozygous reporter (+/r), or homozygous reporter (r/r) mice were treated with 30 mg kg^−1^ haemin and killed 18 h later. All mice were male and aged between 12 and 13 weeks. *B*, pooled liver, lung, or large intestine (LI) whole lysates were prepared and blotted for Hmox1, β‐galactosidase and firefly luciferase. GAPDH was used as a loading control. Molecular weights are noted on the right. The asterisk indicates the slower‐migrating 2A‐tagged form of Hmox1. *C*, levels of hepatic *Hmox1* and *LacZ* mRNA relative to 18s rRNA. Data are presented as arbitrary units and have been transformed so that the median level of *Hmox1* mRNA in WT mice is set to a value of 1. The median level of *LacZ* mRNA in heterozygous reporter mice was set to 1. *D*, *in vivo* bioluminescence images of mice before and after treatment with haemin (hemin). Note that one of the homozygous reporter mice (asterisked) displayed an atypically low luminescence post‐haemin. Data points from this mouse are highlighted in graphs *C*, *E* and *F* by increased transparency. *E*, quantification of images in *D*. *F*, firefly luciferase activity per mg protein was measured in lysates prepared from lung, liver, or LI. Data are presented in arbitrary units. In order to highlight the fold difference in activity between heterozygous and homozygous reporter mice, data have been transformed so that the median activities in organs from heterozygous reporter mice are set to a value of 1.

We created a mouse line in which an open reading frame encoding T2A‐β‐gal‐T2A‐hCG‐T2A‐luciferase was inserted between the penultimate amino acid‐encoding codon and the STOP codon in exon 5 of the *Hmox1* gene (Fig. 1 *A*). T2A peptides promote a phenomenon known as ribosome skipping that allows multiple proteins to be expressed from a single mRNA (de Felipe *et al*. 2006). We therefore expected that four separate polypeptides, Hmox1, β‐gal, hCG and luciferase, would be expressed from this engineered allele. It is worthy of note that the Hmox1 expressed from the reporter allele contains the T2A amino acid sequence at its C‐terminus. The resulting increase in molecular weight allows one to distinguish between Hmox1 expressed from the reporter and wild‐type (WT) alleles.

In order to investigate the level of Hmox1 expression off the engineered gene locus carrying the T2A tag, we treated WT mice, and heterozygous and homozygous reporter mice (i.e. bearing one and two *Hmox1* reporter alleles, respectively) with haemin (hemin), the substrate for and inducer of *Hmox1*. Following *in vivo* luminescence imaging for reporter activity, organs were harvested for protein and mRNA analyses (Fig. 1 *B*–*F*). Three separate peptides from the reporter transcript were detected by blotting liver, lung and large intestine lysates for Hmox1, β‐gal and luciferase proteins (Fig. 1 *B*). Specifically, two bands of the predicted molecular weights for the Hmox1 expressed from the native allele and the T2A‐tagged Hmox1 expressed from the reporter allele were detected (Fig. 1 *B*). Both β‐gal and luciferase were detected as single proteins with higher expression levels in animals homozygous for the reporter allele. We could not detect hCG in any of these samples which we ascribe to the fact that this is a secreted protein. Nevertheless, correct processing of the two polypeptides that flank it, β‐gal and Luc, implies that hCG was also expressed.

### Heterozygous reporter mice do not display a phenotype despite suppressed expression of Hmox1

Although the 2A strategy for the expression of multiple reporters off a single allele worked well, the level of Hmox1 expression from the reporter gene was significantly lower than its WT counterpart (Fig. 1 *B*). We have made a similar observation in a p21 reporter mouse made using a similar strategy (McMahon *et al*. 2016). Reduced expression of Hmox1 protein from the reporter locus can be explained, at least in part, by a reduction in the corresponding mRNA transcript (Fig. 1 *C*). The *Hmox1* TaqMan assay does not distinguish between mRNA from WT and reporter alleles. Bearing this in mind, the fact that heterozygous reporter mice (one WT gene) expressed approximately half the amount of mRNA found in wild‐type mice (two WT genes) implies that transcription of the reporter allele was low. This is also consistent with the finding that the level of *Hmox1* mRNA in homozygous reporter mice was even further reduced (Fig. 1 *C*).

**Figure 2:**
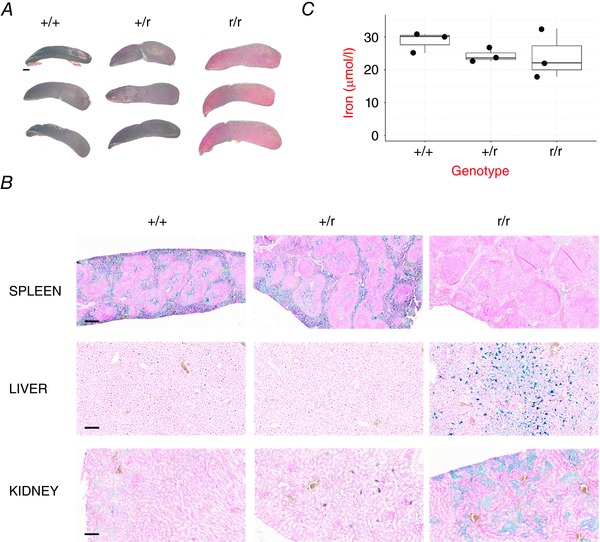
Phenotypic abnormalities in reporter mice Triplicate age‐matched (41 week old) WT (+/+), heterozygous reporter mice (+/r), or homozygous (r/r) reporter mice were killed. *A*, spleens from each animal are shown. Scale bar = 1 mm. *B*, Perls Prussian Blue was used to detect iron‐loading in spleen, liver and kidney sections. Representative images are shown. Scale bar = 250 μm (spleen) or 100 μm (liver and kidney). *C*, iron levels in plasma. The differences in iron levels across genotypes were not significant (one‐way ANOVA).

*Hmox1^−/−^* mice display a number of phenotypes, including an exaggerated response to haemin, splenomegaly that presents with age, a deficit in iron re‐utilization that manifests as an accumulation of iron in the liver and kidney with a corresponding reduction in blood‐borne iron, and almost complete perinatal lethality (Poss & Tonegawa, 1997a, b). Consistent with reduced expression of Hmox1 protein, homozygous reporter mice also exhibited these phenotypes with the exception of perinatal lethality. Examination of 41‐week‐old homozygous reporter mice revealed marked splenomegaly (Fig. 2 *A*). Staining with Perls' Prussian Blue revealed that these same animals also had deposits of iron in their livers and kidneys with a corresponding depletion in splenic iron levels (Fig. 2 *B*). *Hmox1* homozygous reporters also mounted an exaggerated response to haemin as evidenced by two separate findings. First, we used a custom TaqMan assay to detect specifically the transcript produced from the reporter locus. This revealed that after exposure to haemin, two of the three homozygous reporter mice (which bear two reporter alleles) expressed considerably more than double the amount of reporter transcript that would be expected on the basis of levels observed in similarly treated heterozygous reporter mice (that bear a single reporter allele) (Fig. 1 *C*). Second, the same two homozygous reporter mice also displayed aberrantly high *in vivo* (Fig. 1 *D* and *E*) and *ex vivo* (Fig. 1 *F*) luminescent signals after exposure to haemin. The third and final homozygous reporter mouse examined did not display an exaggerated response to haemin by these measures (Fig. 1 *C*–*F*).

It is important to note that the homozygous Hmox1 reporter still expresses some active Hmox1. Consistent with this, whereas the complete knockout of Hmox1 displays almost complete perinatal lethality (Poss & Tonegawa, 1997b), homozygous reporter mice were born, albeit at a sub‐Mendelian frequency (16% observed *vs*. 25% expected, significant at *P* < 0.01 by χ^2^ test), and grew to adulthood. Additionally, there was only a mild and statistically non‐significant decline in blood iron levels (Fig. 2 *C*), in contrast to the marked reduction reported for the complete knock‐out (Poss & Tonegawa, 1997b).

Importantly, heterozygous reporter mice were phenotypically normal; they did not display splenomegaly or altered iron re‐utilization (Fig. 2), and displayed the expected response to haemin (Fig. 1 *B*). The heterozygous reporter mice are therefore suitable for studying normal physiological processes and were used in all subsequent experiments.

### Regulation of the Hmox1 reporter reflects the expression of the wild‐type Hmox 1 allele

We then carried out experiments to establish whether the expression of the reporter proteins faithfully mimicked the expression of the endogenous gene. We anticipated that this would be the case as all the coding and regulatory elements that control Hmox1 expression were retained in the reporter mice. The published literature on the endogenous expression of Hmox1 in mouse tissues is limited, however, as evidenced by the lack of data curated in Mouse Genome Informatics (MGI; http://www.informatics.jax.org). For this reason, we initially studied β‐galactosidase expression patterns in spleen, liver and kidney for which Hmox1 expression data have been reported, as described below.

**Figure 3:**
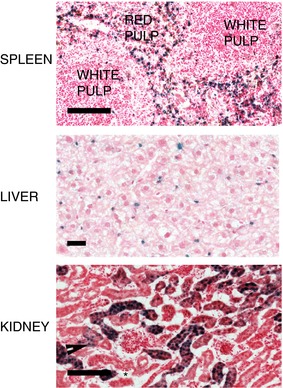
*In situ* β‐galactosidase activity in spleen, liver and kidney of control reporter mice Representative spleen, liver and kidney sections from reporter mice aged between 10 and 14 weeks. Scale bar = 100 μm (spleen), 25 μm (liver), 100 μm (kidney). In the kidney image, the arrowhead indicates a tubule; asterisks indicate glomeruli.

In healthy mice, Hmox1 activity has been reported in splenic and hepatic macrophages/Kupffer cells (Maines, 1997; Ryter *et al*. 2006; Abraham & Kappas, 2008) and in tubular epithelial cells in the kidney (Nath *et al*. 2000, 2001). Our data are consistent with these previous observations. For example, the size and morphology of β‐galactosidase‐positive cells in spleen and liver sections from control reporter mice (Fig. 3) are consistent with their being macrophages. Moreover, positive cells in the spleen were found exclusively in the red pulp, where macrophages reside, and not in the white pulp. In the kidney, positive cells were generally detected in tubules of the cortex (Fig. 3); glomerular epithelial cells tended to be negative for reporter activity (Fig. 3).

**Figure 4:**
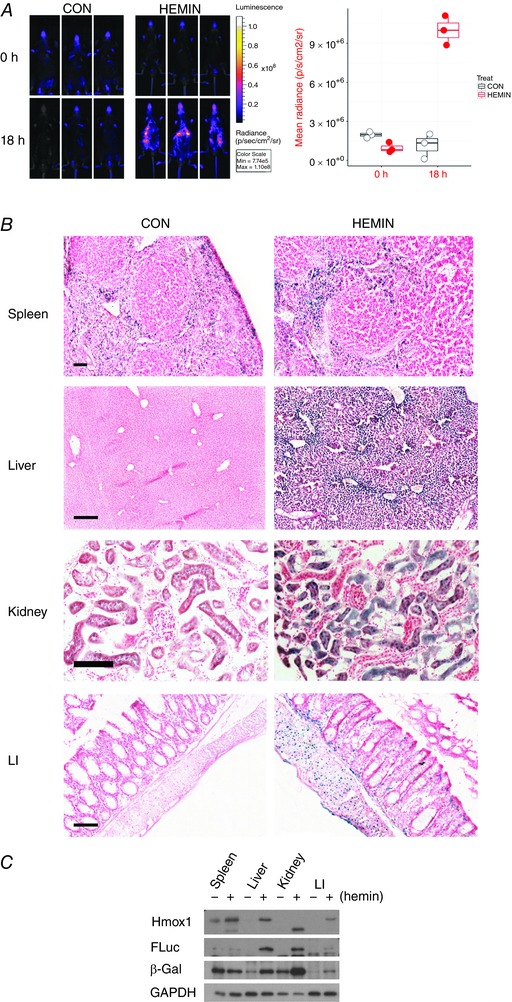
Haemin induces Hmox1 reporters in liver and kidney cells Triplicate reporter mice were treated with 30 mg kg^−1^ haemin and killed 18 h later. All mice were male and aged between 19 and 20 weeks except for one haemin‐treated mouse that was 14 weeks old. *A*, mice were imaged at baseline and again just before necropsy. The accompanying graph quantifies the luminescent signal. The effect of haemin was significant at *P* < 0.0005 (two‐way ANOVA with repeated measures). *B*, spleen, liver, kidney and large intestine (LI) sections from control and haemin‐treated mice were *in situ* β‐galactosidase stained. Representative images are shown. Scale bar = 250 μm except LI (100 μm) and kidney (50 μm). *C*, pooled organ lysates (*n* = 3) were probed for the indicated proteins. GAPDH was used as a loading control. The Hmox1 present in the treated kidney sample migrates more rapidly than expected and probably represents a partially proteolysed product.

Exposure of mice to haemin is reported to induce *Hmox1* in hepatocytes, renal tubular cells and macrophages (Nath *et al*. 2000; Ryter *et al*. 2006). Induction of Hmox1 was clearly visible by whole‐body *in vivo* imaging 18 h after haemin administration to reporter mice (Fig. 4 *A*), although it was not possible to determine the affected organs from the observed luminescence patterns. However, β‐galactosidase staining of organ sections post‐mortem demonstrated that reporter activity was highly induced in hepatocytes. The response showed distinct zonation being particularly prevalent in centrilobular hepatocytes and lowest in periportal regions (Fig. 4 *B*), as previously reported (Bauer & Bauer, 2002). Reporter activity was also induced to a lesser extent in cells of the renal tubule but not in glomerular cells (Fig. 4 *B*). Moreover, the staining pattern in the spleen was unchanged after this treatment (Fig. 4 *B*). Our data are therefore consistent with the earlier literature reports – but they also extend them. For example, a marked induction of reporter activity was observed using our model in the smooth muscle cells lining the gastrointestinal tract (Fig. 4 *B*). This suggested a previously unsuspected induction by haemin of Hmox1 in the large intestine, which was confirmed by immunoblotting (Fig. 4 *C*). Quantitatively, these immunoblotting data revealed a good correlation between Hmox1 and β‐galactosidase expression levels. In the case of luciferase, its expression was less well correlated with Hmox1 and β‐galactosidase, primarily due to lower than expected levels in the spleen. These observations highlight the fact that, as with any reporter strategy, differences in protein stability are likely to prevent a perfect match between target and reporter protein levels.

**Figure 5:**
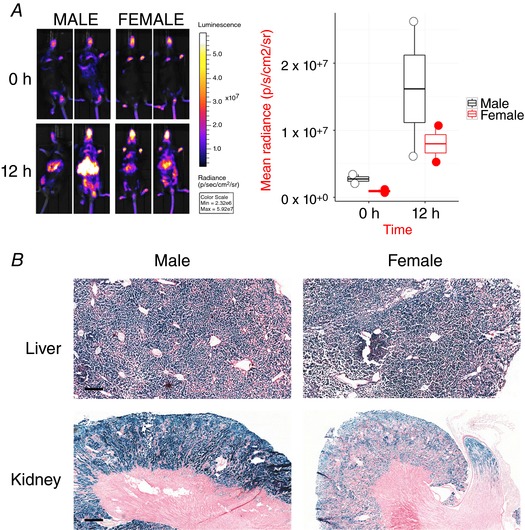
Cadmium chloride causes sexually dimorphic induction of Hmox1 reporter signals in liver and kidney Two male and two female mice were treated with 4 mg kg^−1^ cadmium chloride (Cd). *A*, bioluminescent images were obtained both before and 12 h after exposure to Cd. Luminescence is quantified in the accompanying graph. *B*, liver and kidney sections were stained for β‐galactosidase activity. Representative images are shown. Scale bar = 250 μm (liver) and 500 μm (kidney).

Finally, cadmium is a nephro‐ and hepato‐toxin that potently induces Hmox1 activity in both liver and kidney (Iwahara *et al*. 1995; Ossola & Tomaro, 1995; Sikorski *et al*. 2006). A very marked increase in luminescence from our model was observed upon treatment with this chemical (Fig. 5 *A*). The pattern of luminescence suggested the liver and kidneys were the predominant organs contributing to this signal. Subsequent *in situ* staining confirmed that β‐galactosidase activity was profoundly induced in hepatocytes, with only limited evidence of zonation in the response (Fig. 5 *B*). In addition, there was a striking demarcation of response in the kidney with significant induction of reporter activity in the cortex that was absent from the medullar region (Fig. 5 *B*); the cortical response occurred both in tubular and glomerular cells.

The above data demonstrate that the reporter system is a *bona fide* marker for Hmox1 protein expression and is highly inducible in multiple organs of the mouse.

### Novel patterns of Hmox1 expression identified through high‐resolution reporter analysis

**Figure 6:**
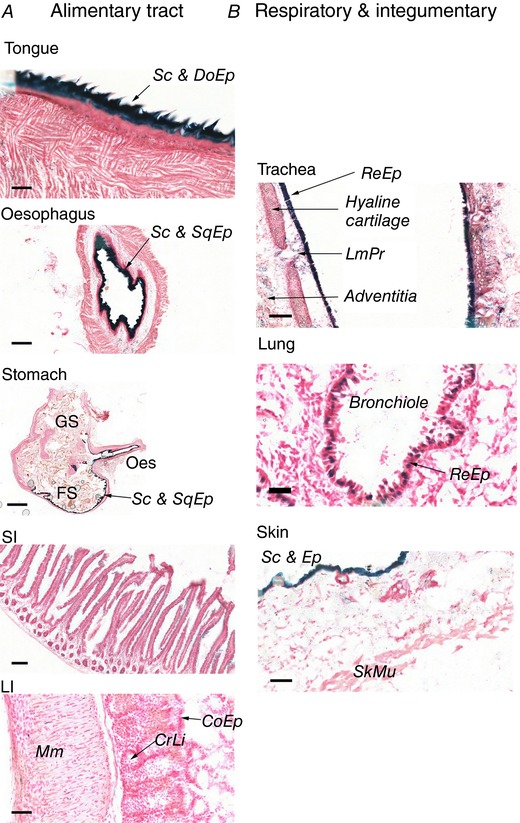
*In situ* β‐galactosidase activity is found in barrier tissues throughout the gastrointestinal, respiratory and integumentary systems Representative images of organ sections from untreated mice. At least three mice were examined per organ. *A*, gastrointestinal tract. *B*, respiratory and integumentary system. Scale bar = 100 μm (tongue, small intestine (SI)), 250 μm (oesophagus, stomach), 50 μm (LI, trachea, skin), 25 μm (lung). Sc: stratum corneum; Ep: epithelium; CoEp: columnar epithelium; DoEp: dorsal epithelium; ReEp: respiratory epithelium; SqEp: squamous epithelium; CrLi: crypts of Lieberkuhn; LmPr: lamina propria; Mm: muscularis mucosa; SkMu: skeletal muscle; GS: glandular stomach; FS: forestomach; Oes: oesophagus.

**Figure 7:**
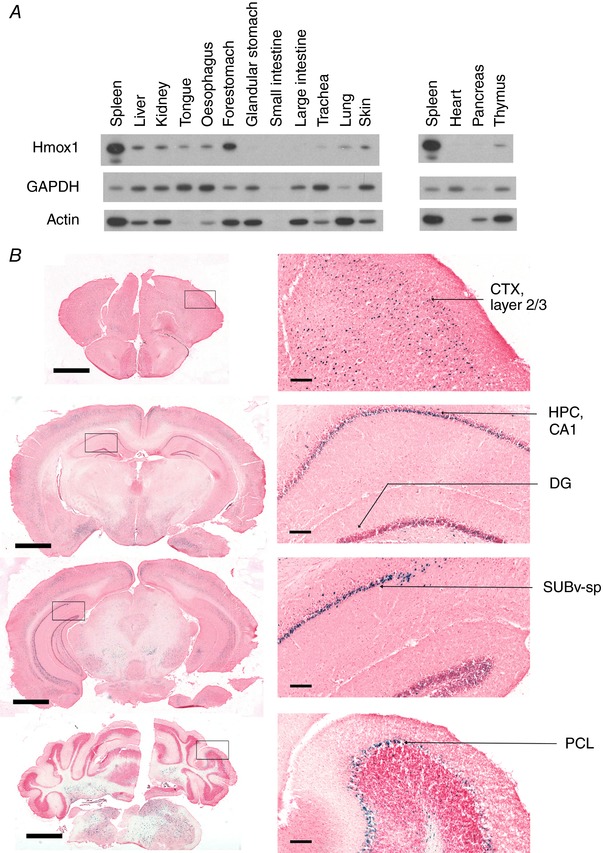
β‐Galactosidase staining of brain sections from an untreated Hmox1 reporter mouse *A*, pooled organ lysates (*n* = 4) from untreated WT mice were probed for Hmox1. Equal amounts of proteins were run in each lane. There is no perfect loading control for this experiment; for example, GAPDH and actin signals were uncorrelated and both varied across organs due to differences in expression levels rather than uneven loading. Due to the number of organs examined, two gels were run and the same spleen sample was run on both. *B*, brain sections were prepared and stained as described in Methods. Scale bar = 1000 μm (main images) or 50 μm (insets). Abbreviations: CTX, cortex; HPC, hippocampus proper; DG, dentate gyrus; SUBv‐sp, subiculum, ventral part, pyramidal layer; PCL, Purkinje cell layer.

To extend the currently limited understanding of Hmox1 expression patterns in mice we performed β‐galactosidase staining on a comprehensive selection of additional mouse organs (Figs 6 and 7). Interestingly, reporter protein was expressed at high levels in those barrier tissues at the interface between the internal and external environment that serve to provide mechanical and chemical protection to the organism from the environment and against oxidative stress.

In the alimentary tract, reporter expression was expressed solely in the stratified, cornified, epithelial layer that extends from the tongue, through the oesophagus, and down to the juncture between the fore‐ and glandular stomach (Fig. 6 *A*). From this juncture onwards, where the structure and function of the epithelial lining changes from stratified, with a protective function to columnar cells, specialized for adsorption of nutrients, reporter activity was no longer observed (Fig. 6 *A*). In the respiratory tract, the protective lining of the trachea and bronchioles were highly and modestly positive, respectively (Fig. 6 *B*). Positive cells are also found in some unidentified cell types within the loose connective tissue (lamina propria and adventitia) layers of the trachea. Finally, the stratified, cornified epithelial layer covering the skin, a major point of defence against the environment, was highly positive for reporter activity (Fig. 6 *B*).

Based on these data we tested whether endogenous Hmox1 could be detected in multiple barrier tissues by western blotting. Although this approach only provides an average measure of Hmox1 across all cell types, the results confirmed that the respiratory system and skin of healthy mice expressed significant amounts of Hmox1 (Fig. 7 *A*). It was striking that the expression pattern of Hmox1 throughout the alimentary tract confirmed our predictions in all important respects; tongue, oesophagus and forestomach were positive for Hmox1 whereas the glandular stomach, and small and large intestines were essentially negative (Fig. 7 *A*). The above data demonstrate the power of the reporter system to chart target protein expression in a *bone fide* manner.

We also examined reporter expression in an additional set of internal organs. The heart and pancreas were reporter negative in healthy animals (data not shown), a conclusion that is corroborated by the western blotting data of Fig. 7 *A*. The thymus was also reporter negative save for a sparse subpopulation of unidentified medullary cells (data not shown). In the anatomically complex brain, Hmox1 was only expressed in a limited number of regions (Fig. 7 *B*). Typically, a scattering of positive pyramidal neurons was found in layers 2/3 of the cortex. Some positivity was also observed in the CA1 field of the hippocampus. The most striking findings in the brain were that the pyramidal layer in the ventral part of the subiculum was highly positive, as was the Purkinje cell layer in the cerebellum.

### Inflammation and oxidative stress induce reporter expression

**Figure 8:**
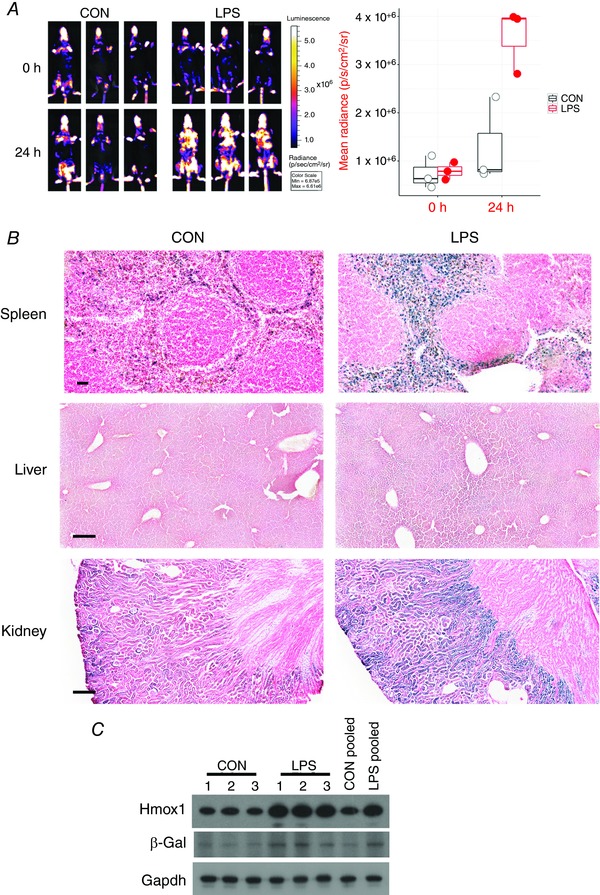
LPS induces reporter activity in spleen and kidney, and to a lesser extent in liver Triplicate reporter mice were treated with vehicle (PBS) or 1 mg kg^−1^ LPS. Two male and one female mouse were used per treatment group. All mice were aged 18 weeks. *A*, mice were imaged for bioluminescence at baseline and just before necropsy (24 h post‐treatment). Note that a compressed scale is used in this image compared to others presented in this paper. Quantification of images is presented in the accompanying graph. Effect of LPS was significant at *P* < 0.002 (two‐way ANOVA with repeated measures). *B*, all livers and kidneys were stained for β‐galactosidase activity. Representative images are shown. Scale bar = 250 μm. *C*, individual and pooled hepatic lysates were blotted for Hmox1 and β‐galactosidase protein. GAPDH was used as a loading control.

**Figure 9:**
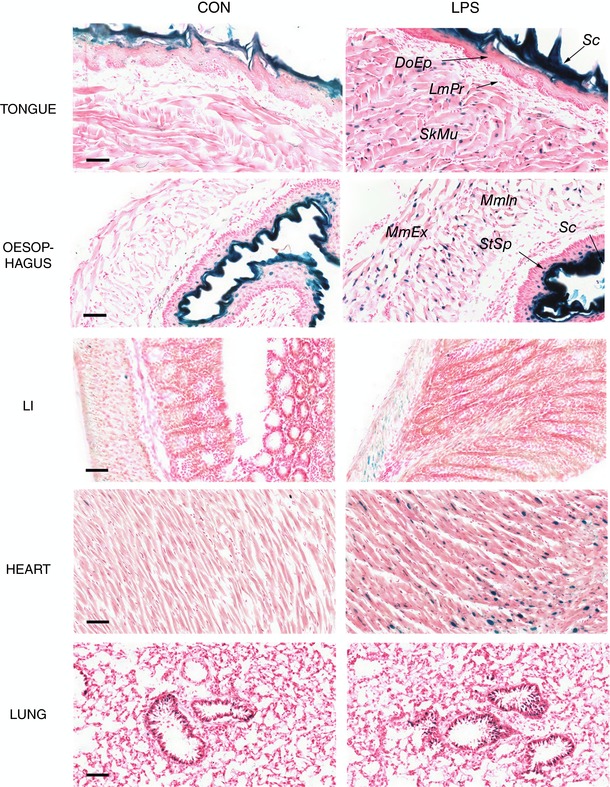
*In situ* β‐galactosidase activity in multiple organs from LPS‐treated reporter mice All tongue, oesophagus, LI, heart and lung samples from the control and LPS‐treated mice described in Fig. 8 were stained for β‐galactosidase activity. Representative images are shown. Scale bar = 50 μm. Sc: stratum corneum; DoEp: dorsal epithelium; LmPr: lamina propria; SkMu: skeletal muscle; StSp: stratum spinosum; MmIn: muscularis interna; MmEx: muscularis externa.

The major pathophysiological states leading to increased expression of Hmox1 are inflammation and oxidative stress (Soares & Bach, 2009; Gozzelino *et al*. 2010). We investigated Hmox1 induction by inflammation by exposing reporter mice to LPS (Figs 8 and 9). A statistically significant increase in luminescence from reporter mice was observed after LPS treatment and the location of the signal on the torso suggested that spleen and kidney played a prominent role in this response (Fig. 8 *A*). Subsequent β‐galactosidase staining of sections confirmed this conclusion and revealed that the induction in these two organs was largely restricted to macrophages (spleen) and tubular epithelial cells (kidney) (Fig. 8 *B*).

It has been reported that inflammation causes a transient induction of *Hmox1* mRNA in hepatocytes as part of the acute phase response (Rizzardini *et al*. 1993), although the increase in protein is much more modest (Bauer, 1998; Carchman *et al*. 2011). Consistent with these data, we only observed slight increases in hepatic levels of both Hmox1 and β‐galactosidase proteins by western blotting lysates prepared from reporter mice 24 h after exposure to LPS (Fig. 8 *C*); moreover, there was no obvious increase in β‐galactosidase staining in liver sections from LPS‐treated mice compared to control mice (Fig. 8 *B*).

A further interesting novel observation was that LPS activated Hmox1 expression in muscle cells in multiple anatomical locations, including the tongue, oesophagus and large intestine, as inferred from β‐galactosidase staining patterns (Fig. 9). In the tongue, the induction of Hmox1 occurred in skeletal muscle cells. In the oesophagus, the induction involved smooth muscle cells of the muscularis interna and externa. The muscular tunic of the large intestine displayed a weak β‐galactosidase signal in treated mice. Finally, cardiomyocytes also responded to LPS by inducing β‐galactosidase (Fig. 9). Muscle cells therefore appear to be a major site of Hmox1 induction, which is supported by our earlier observation of haemin‐elicited increase in β‐galactosidase activity in the smooth muscle cell layers surrounding the large intestine (Fig. 4 *B*).

**Figure 10:**
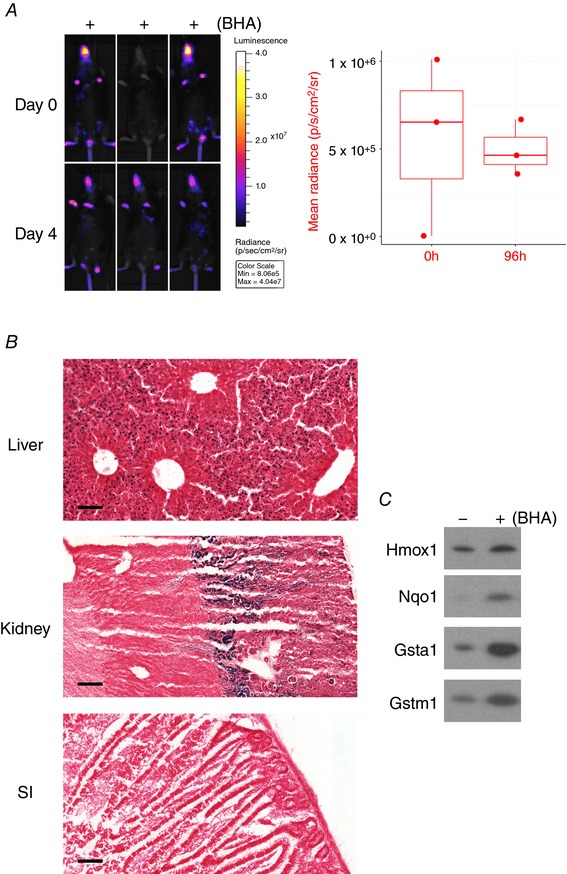
BHA induces reporter activity in hepatocytes and kidney tubules *A* and *B*, triplicate female reporter mice aged between 10 and 14 weeks were administered BHA in the RM1 foodstuff at a final concentration of 0.5% (w/w) for 4 days. *A*, mice were imaged for bioluminescence at baseline and just before necropsy. Quantification of images is provided in the accompanying graph. Effect of BHA was not significant (Student's *t* test with repeated measures). *B*, liver, kidney and small intestine (SI) sections were stained for β‐galactosidase. Scale bar = 50 μm. *C*, hepatic lysates prepared from female control mice or mice administered BHA in foodstuff for 4 days were probed for Hmox1, Nqo1, Gsta1 and Gstm1.

To establish the sensitivity of the reporter to oxidative stress, mice were treated with butylated hydroxyanisole (BHA), which is metabolized *in vivo* to 2‐*tert*‐butyl‐1,4‐benzoquinone, an electrophilic metabolite that is a potent activator of Nrf2 (Abiko & Kumagai, 2013). We previously reported that exposure of mice to 0.5% (w/w) BHA in their foodstuff led to substantial Nrf2‐dependent increases in expression of various members of the glutathione *S*‐transferase (Gst) family and NAD(P)H:quinone oxidoreductase 1 (Nqo1) in both liver (Chanas *et al*. 2002) and small intestine (McMahon *et al*. 2001). On this basis, we anticipated that *Hmox1* would be similarly induced and indeed although bioluminescence did not increase in reporter mice administered BHA for 4 days (Fig. 10 *A*), they did display a β‐galactosidase signal in mid‐zonal hepatocytes (Fig. 10 *B*). An unusual pattern of reporter induction was also apparent in the kidney, where β‐galactosidase activity was increased exclusively in the juxtamedullary region of the cortex (Fig. 10 *B*). Although these data confirm that Hmox1 is inducible *in vivo* by BHA, the level of hepatic reporter expression was modest at the 4 day time point and we failed unexpectedly to detect any reporter signal in the small intestine (Fig. 10 *B*). One difference between *Hmox1* and *Gst/Nqo1* genes is that the former – but not the latter – is one of only a small subset of Nrf2‐regulated genes that is also negatively regulated by Bach1. As Bach1 rapidly attenuates Nrf2‐driven responses (Jyrkkänen *et al*. 2011; Fuse *et al*. 2015), we hypothesize that the modest Hmox1 reporter responses observed after 4 days of BHA treatment reflect a more transient induction of *Hmox1* compared with Nrf2‐regulated *Gst* and *Nqo1* genes. Consistent with such an interpretation, hepatic levels of Gsta1, Gstm1 and Nqo1 were significantly elevated in mice treated with BHA for 4 days, whereas only a very slight increase in Hmox1 protein was observed at this time point (Fig. 10 *C*). Further research is required to establish whether Hmox1 induction is more pronounced at earlier time points.

### Measurement of the attenuation of oxidative stress induced by acetaminophen with *N*‐ acetyl cysteine

**Figure 11:**
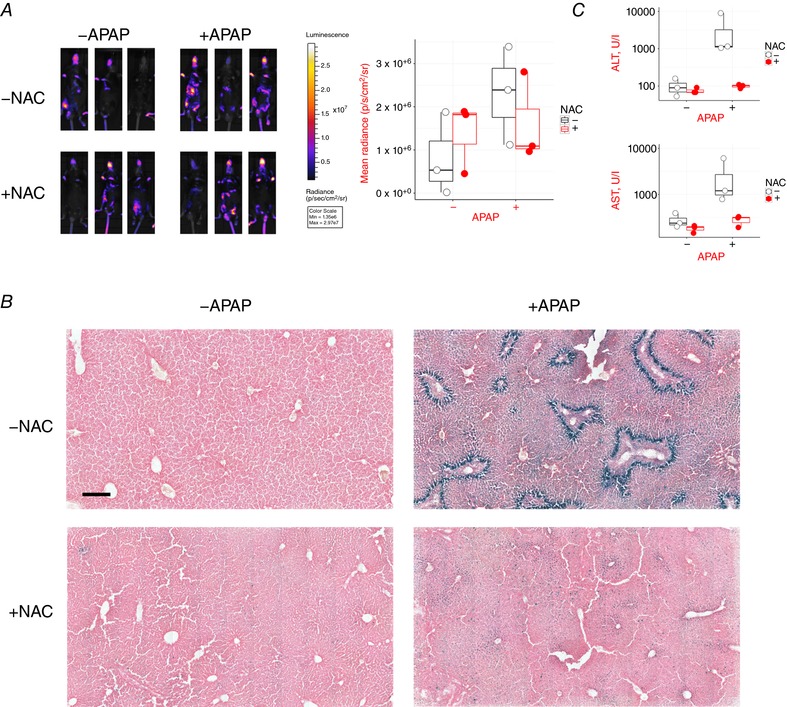
APAP induces reporter activity in the liver that is mitigated by the antidote NAC Triplicate reporter mice were treated with vehicle, 300 mg kg^−1^ APAP, 300 mg kg^−1^ NAC, or a combination of 300 mg kg^−1^ APAP + 300 mg kg^−1^ NAC, and necropsied 24 h later. Each treatment group contained two male and one female mouse. Mice were between 9 and 10 weeks of age. *A*, mice were imaged for bioluminescence at baseline and just before necropsy. Quantification of images is provided in the accompanying graph. Neither of the effects of APAP or NAC on bioluminescence were statistically significant (two‐way ANOVA). *B*, all livers were sectioned and stained for β‐galactosidase activity. Representative images are shown. Scale bar = 250 μm. *C*, ALT and AST activities in plasma obtained at necropsy. Two‐way ANOVA confirmed that APAP significantly increased both activities, which was prevented by co‐administration of NAC. ALT: *P* < 1 × 10^−7^ for effect of APAP; *P* < 2 × 10^−7^ for APAC × NAC interaction. AST: *P* < 4 × 10^−4^ for effect of APAP; *P* < 2 × 10^−4^ for APAC × NAC interaction.

To further exemplify the use of the reporter mice to detect oxidative stress and to investigate the potential of the reporter system to identify agents which prevent oxidative stress‐induced damage, we treated mice with a toxic dose of acetaminophen (APAP) plus or minus the antidote to APAP toxicity, *N*‐acetyl cysteine (NAC) (Black, 1984; Nelson, 1990). APAP is metabolized in the liver to a highly thiol‐active imido‐quinone in hepatocytes which causes glutathione depletion and oxidative stress (McGarry *et al*. 2015). APAP mildly increased the bioluminescent signal from the reporter mouse (Fig. 11 *A*), although the trend was not statistically significant by two‐way ANOVA. However, it markedly induced reporter activity in hepatocytes as measured by β‐galactosidase staining (Fig. 11 *B*). β‐Galactosidase staining was observed as a ring of activity surrounding a necrotic area in the pericentral hepatocytes (McGarry *et al*. 2015). Importantly, co‐administration of NAC markedly reduced both the β‐galactosidase signal and the hepatotoxicity as measured by alanine aminotransferase (ALT) and aspartate aminotransferase (AST) liver function tests (Fig. 11 *C*). Interestingly, in spite of the lack of damage surviving cells displayed a mild induction of Hmox1, assessed by β‐galactosidase activity (Fig. 11 *B*), indicating that some degree of oxidative stress was still occurring.

### Ionizing radiation does not induce acute oxidative stress

**Figure 12:**
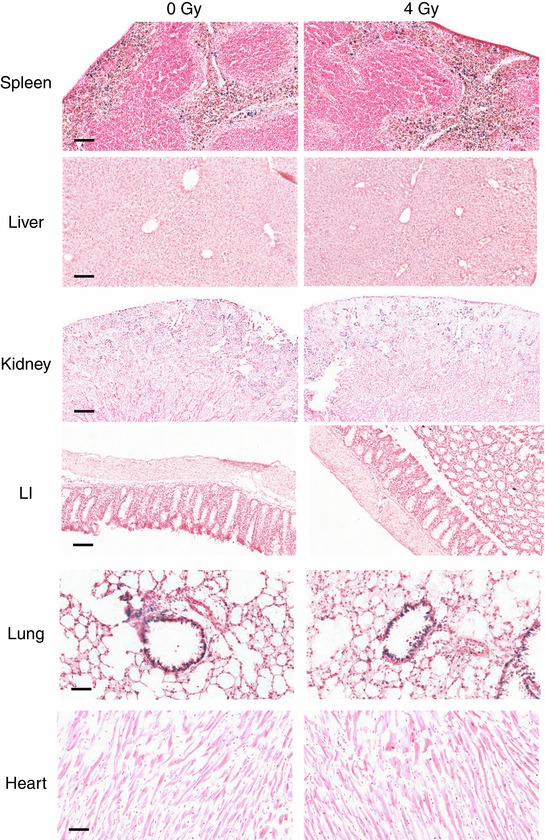
Ionizing radiation does not alter bioluminescence in reporter mice Triplicate reporter mice were sham‐irradiated (0 Gy) or exposed to 4 Gy of IR. All mice were female and aged between 17 and 21 weeks. Mice were killed 24 h post‐irradiation and spleen, liver, kidney, LI, lung and heart sections were stained for β‐galactosidase activity. Scale bar = 100 μm (spleen, liver, LI), 250 μm (kidney), and 50 μm (lung and heart).

**Figure 13:**
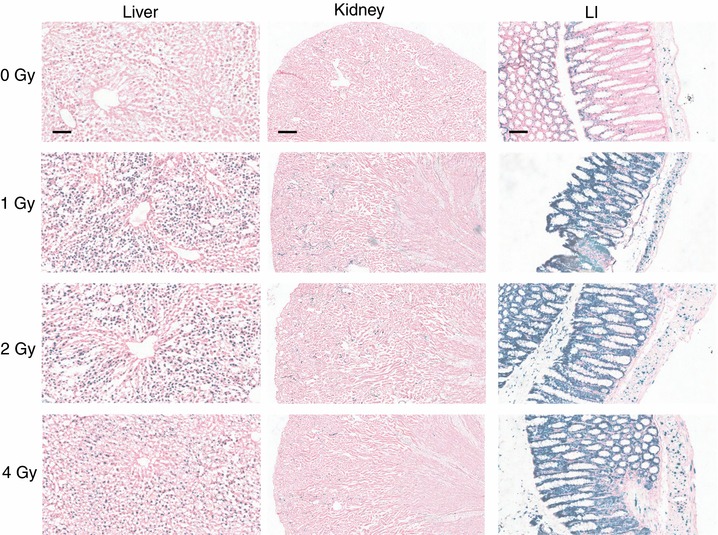
Ionizing radiation induces p21 in liver and LI, even at low doses Triplicate p21 reporter mice were sham‐irradiated or exposed to 1‐, 2‐, or 4 Gy of IR. All mice were female and aged between 16 and 21 weeks. Mice were killed 24 h post‐irradiation and stained for β‐galactosidase activity. Representative images are shown. Scale bar = 100 μm (liver and LI) or 250 μm (kidney).

The toxic effects of ionizing radiation (IR) have been linked to the induction of oxidative stress although it remains unclear whether this is an acute effect of IR or a delayed secondary response (Azzam *et al*. 2012). To investigate this issue *in vivo*, Hmox1 reporter mice were exposed to 4 Gy of IR. Interestingly, this treatment did not induce any detectable increase in oxidative stress/reporter activity in any of the tissues measured (β‐galactosidase activity – Fig. 12; bioluminescence – data not shown). In contrast, this treatment caused a profound induction of the DNA damage‐inducible gene *p21* in p21 reporter mice in liver and intestine (Fig. 13). Indeed, in the IR‐sensitive large intestine, the signal was already saturated even at the lowest dose of IR tested (1 Gy). These data suggest that oxidative stress is not an acute effect of ionizing radiation *in vivo*, at least at the doses tested.

## Discussion

We describe a method for assessing Hmox1 expression across all cell lineages *in vivo* in mice. The model was developed to allow expression to be monitored non‐invasively over extended periods of time (bioluminescence imaging) as well as at single‐cell resolution post‐mortem (β‐galactosidase staining). The goal of this study was to validate and rigorously assess the performance of the model, provide a blueprint for Hmox1 expression in healthy mice, and exemplify its use in measuring cellular toxicity.

We have demonstrated that the use of viral 2A sequences can be used to express multiple reporter proteins, as well as the endogenous gene, from the endogenous *Hmox1* gene locus. The strategy worked sufficiently well to generate a highly sensitive reporter read out which faithfully reflected the expression of the endogenous gene following a variety of different animal treatments. Although the reporter system also contained the coding sequence for the secretable marker hCG we were unable to detect this protein in any of the blood samples studied, presumably because it was rapidly excreted (data not shown).

The expression of Hmox1 protein from the reporter allele was, however, significantly lower than from the unmodified endogenous gene. This appeared to be due to a reduced rate of transcription or an increased rate of mRNA degradation. The markedly reduced Hmox1 expression was also reflected in that mice homozygous for the reporter exhibited a number of phenotypes similar to *Hmox1* null animals (Poss & Tonegawa, 1997a, b). However, the fact that some Hmox1 protein could be detected and that the mice did not exhibit the neonatal mortality seen in animals lacking Hmox1 (Poss & Tonegawa, 1997b) provides evidence that this allele is still functional. It is important to note that mice heterozygous for the reporter did not display any adverse phenotype and are therefore suitable for investigating normal Hmox1 physiology as well as providing a sensor of physiological change. This conclusion is supported by the finding that many healthy humans are predicted to possess only one functional *Hmox1* allele (ExAC consortium: http://exac.broadinstitute.org/gene/ENSG00000100292). It is worthy of note that the homozygous mice may also prove very useful for studying Hmox1 functions.

Gene expression patterns provide important insights into physiological functions. Through the use of the reporter system two new findings about the expression of Hmox1 are particularly worthy of note. First, healthy mice express considerable amounts of Hmox1 in barrier tissues lining the alimentary, respiratory and integumentary systems. Much of this positive barrier tissue is composed of stratified, keratinized epithelium, including the stratum corneum (Sc) of the skin. Nrf2, a transcription factor known to regulate *Hmox1*, drives expression of some Sc‐restricted genes, such as *secretory leukocyte protease inhibitor* and *Small proline‐rich repeat domain 2d* (Schäfer *et al*. 2012, 2014) and may therefore be responsible for expression of Hmox1 in this location also.

Our finding suggests that Hmox1 may play an important role in the physiology of Sc and protective stratified epithelia more generally. The Sc for example is composed of corneocytes embedded in lipids that are susceptible to peroxidation arising from exposure to e.g. UV irradiation (Gibbs & Norval, 2011). One possibility therefore is that Hmox1 serves a protective role by catalysing the conversion of haem to the lipid‐soluble anti‐oxidant bilirubin to neutralize this peroxidation. Indeed, bilirubin has been reported to be produced in the skin specifically in the Sc (Numata *et al*. 2009). On this basis, Hmox1 might be responsible for *in situ* production of anti‐oxidants at barrier tissues. This leads to the possibility that Hmox1 deficiency might contribute to skin and other diseases as a consequence of barrier dysfunction. It is important to note that although corneocytes are traditionally described as dead cells, they are capable of engaging in biochemistry and can support enzymatic reactions. For example, citrullination of filaggrin takes place in these cells (Sandilands *et al*. 2009).

A second intriguing finding was that Hmox1 is highly inducible in the muscle cells of mice exposed to environmental stress. This was observed in smooth muscle cells lining the large intestine following haemin exposure and in muscle tissue of several organs in response to pro‐inflammatory signals. Why muscle cells are particularly prone to inducing Hmox1 is unclear. However, as these cells are highly metabolically active they may be particularly sensitive to oxidative stress induced by cytokines or the pro‐oxidant effects resulting from the dysregulation of haem homeostasis. In this context, it is also intriguing to note that muscle cells specifically express myoglobin, the second most abundant haem protein in the body. It is possible therefore that catabolism of haem to carbon monoxide and bilirubin by Hmox1 may represent an important cytoprotective pathway in this cell type.

In addition to using the reporter system to dissect the biochemical role of Hmox1, our model provides a powerful approach to study pathways of chemical toxicity or perturbations in physiological processes involving oxidative stress, which result in human diseases such as neurodegeneration. We have exemplified this use by studying APAP poisoning and its reversal with NAC where a striking reduction in reporter activity was measured in line with the reduction in toxicity.

It is well established that IR damages DNA directly through energetic disruption of DNA integrity but also indirectly through production of hydroxyl radicals via hydrolysis of water. This has led to the widespread belief that IR causes oxidative stress (Simone *et al*. 2009). In a further application of the Hmox1 reporter model, we investigated this issue while measuring DNA damage in parallel using our recently described p21 DNA damage reporter (McMahon *et al*. 2016). Whereas marked induction of p21 was observed in multiple tissues, no induction of Hmox1 was seen in any of the tissues studied. This important finding suggests that the level of hydroxyl radicals required to cause measurable DNA damage is not sufficient to induce oxidative stress leading to protein and lipid damage and induction of Hmox1. These data demonstrate the power of using the two models together to delineate the mechanism of cell toxicity. It is possible that higher doses of – or chronic exposure to – ionizing radiation may elicit a measurable degree of oxidative stress.

In summary, we report the generation of a novel reporter system which allows both the physiological role of Hmox1 to be delineated as well as providing a sensitive system for establishing the role of oxidative stress and/or inflammation in chemical‐induced toxicity or in the pathogenesis of a degenerative disease. We believe the latter possibility is of particular promise because of the paucity of tools to study the pathogenesis of these diseases and tools to measure the effectiveness of therapeutic interventions. The use of a combination of the Hmox1 reporter system together with our recently described DNA damage reporter further strengthens this experimental approach.

## Additional information

### Competing interests

The authors do not have competing financial interests.

### Author contributions

C.R.W., C.J.H. and M.M. conceived and designed the work. All authors analysed data and contributed to writing the manuscript. All authors have approved the final version of the manuscript and agree to be accountable for all aspects of the work. All persons designated as authors qualify for authorship and all who qualify for authorship have been listed.

### Funding

This work was supported by a European Research Council Advanced Investigator Award (number 294533) and a CRUK Programme Grant (C4639/A10822), both to C.R.W.
